# Discrepancies between observed data and predictions from mathematical modelling of the impact of screening interventions on *Chlamydia trachomatis* prevalence

**DOI:** 10.1038/s41598-019-44003-x

**Published:** 2019-05-17

**Authors:** Joost Smid, Christian L. Althaus, Nicola Low

**Affiliations:** Institute of Social and Preventive Medicine (ISPM), University of Bern, Bern, Switzerland

**Keywords:** Infectious diseases, Preventive medicine

## Abstract

Mathematical modelling studies of *C*. *trachomatis* transmission predict that interventions to screen and treat chlamydia infection will reduce prevalence to a greater degree than that observed in empirical population-based studies. We investigated two factors that might explain this discrepancy: partial immunity after natural infection clearance and differential screening coverage according to infection risk. We used four variants of a compartmental model for heterosexual *C*. *trachomatis* transmission, parameterized using data from England about sexual behaviour, *C*. *trachomatis* testing, diagnosis and prevalence, and Markov Chain Monte Carlo methods for statistical inference. In our baseline scenario, a model in which partial immunity follows natural infection clearance and the proportion of tests done in chlamydia-infected people decreases over time fitted the data best. The model predicts that partial immunity reduced susceptibility to reinfection by 68% (95% Bayesian credible interval 46–87%). The estimated screening rate was 4.3 (2.2–6.6) times higher for infected than for uninfected women in 2000, decreasing to 2.1 (1.4–2.9) in 2011. Despite incorporation of these factors, the model still predicted a marked decline in *C*. *trachomatis* prevalence. To reduce the gap between modelling and data, advances are needed in knowledge about factors influencing the coverage of chlamydia screening, the immunology of *C*. *trachomatis* and changes in *C*. *trachomatis* prevalence at the population level.

## Introduction

There is ongoing debate about the evidence to support screening for *Chlamydia trachomatis* (chlamydia) infection to reduce prevalence^1–3^. *C*. *trachomatis* is the most commonly reported bacterial sexually transmitted infection (STI) in high-income countries; in 2016, about 128,000 cases of *C*. *trachomatis* were diagnosed among young people aged 15–24 years in England^4^ and over 1 million in the United States of America^5^. *C*. *trachomatis* can cause pelvic inflammatory disease (PID) in women, which can lead to ectopic pregnancy and tubal factor infertility^6^. However, *C*. *trachomatis* infection is often asymptomatic, so screening has been promoted to detect and treat asymptomatic infection to prevent reproductive tract morbidity and reduce transmission. In England, screening for chlamydia increased considerably with the National Chlamydia Screening Programme (NCSP) in 2003. Through the NCSP, free opportunistic screening is offered to sexually active women and men under 25 years of age, with nationwide roll-out achieved in 2008. Testing coverage in young women increased from 4% in 2000 to 35% in 2012^7^. However, chlamydia prevalence, estimated in two cross-sectional population-based British National Surveys of Sexual Attitudes and Lifestyles (Natsal) was similar in adults aged 18 to 24 years; in 1999–2001 (Natsal-2) women 3.1% (95% confidence interval, CI 1.8–5.2) and men 2.9% (1.3–6.3) and in 2010–2012 (Natsal-3), women 3.2% (2.2–4.6) and men 2.6% (1.7–4.0)^8,9^.

Transmission dynamic modelling studies predict that screening at levels achieved by the NCSP in England should reduce *C*. *trachomatis* prevalence^10^. These modelling studies describe sexual networks and the dynamics of infection transmission using different structures and levels of complexity^11–14^. In a simpler model, without detailed sexual behaviour, Lewis and White inferred changes in *C*. *trachomatis* prevalence and incidence using time-series data about chlamydia testing and diagnoses in England between 2000–2015^7,15^. Their model output proposed that prevalence had declined as chlamydia testing increased and increased as testing levels fell. An assumption in these models, irrespective of structure, is that amongst people without symptoms suggestive of infection, testing for chlamydia is not influenced by the underlying risk of infection in the screened population. In reality, the NCSP in England primarily tests people at an increased risk of chlamydia and the dataset includes both screening tests done amongst asymptomatic individuals and tests done to diagnose symptomatic infection^16^. Further, with increasing chlamydia test coverage and falling test positivity^7^, if prevalence stayed at similar levels then the proportion of tests done in those at lower risk of infection must have increased.

Immunity also affects the model-predicted impact of screening if treatment inhibits the development of immunity otherwise experienced after natural clearance of infection^17,18^. In a model accounting for immunity, individuals that clear infection naturally are temporarily or partially protected from the force of infection which results in a less rapid turnover of *C*. *trachomatis* within the modelled population. This reduces the predicted impact of screening. Immunity is often not included as part of the natural history in *C*. *trachomatis* transmission models^10^ and, in practice, not much is known about the strength and duration of immunity. Clinical and animal studies suggest that immunity is probably partial instead of fully protective^19,20^. A recent modelling study found that a strong partial immunity reducing susceptibility to reinfection by 70–90% should be present to explain observed prevalence patterns^21^. In another modelling study, Johnson and colleagues estimated a period of immunity of 6–17 years by fitting their model to chlamydia notification data, but they assumed that immunity was fully protective^18^.

In this paper, we use data about sexual behaviour and the prevalence of *C*. *trachomatis* in the general population of Great Britain from Natsal-2 and Natsal-3 and time-series data about chlamydia testing and diagnoses in England and across the same time period. Using a *C*. *trachomatis* transmission model, we investigated two hypotheses about factors that might attenuate the effects of a chlamydia screening intervention: the existence of long-lasting partial immunity; and differential chlamydia test coverage according to the risk of being infected.

## Methods

### Data

Natsal is conducted by face-to-face and computer-assisted questionnaire amongst a stratified random sample of the resident population of Great Britain at ten year intervals since 1990. Natsal-2 includes data about 12,110 respondents aged 16–44 years from 1999–2001, and Natsal-3 includes data about 15,162 respondents aged 16–74 years from 2010–2012^22,23^. Starting with Natsal-2, a random sample of participants who have ever had sexual intercourse has been invited to provide a first catch urine sample, which is tested for the presence *of C*. *trachomatis* using a nucleic acid amplification test (3,608 respondents aged 18–44 years in Natsal-2 and a 4,550 respondents aged 16–44 years in Natsal-3). We used data from heterosexual respondents between 16–44 years about the number of new heterosexual partners in the last year, the respondent’s age at first heterosexual intercourse and the respondent’s age and partner ages at the time of first sexual intercourse with the first, second and third most recent heterosexual partner. We aggregated these data for both surveys because there were no significant differences for these variables between the datasets^22^.

We used estimates for the numbers of chlamydia tests and diagnoses from 2000 to 2011 in England from Chandra and colleagues^7^. The authors collated data from specialist and non-specialist settings in England, which include all NCSP tests from 2003 onwards. They applied assumptions to fill gaps in different data sources from 2000 to 2007 and construct plausible minimum and maximum estimates for the numbers of tests and diagnoses each year. The data are presented for men and women separately in five-year age groups: 15–19, 20–24, 25–34 and 35–44 year olds as rates per 100 persons, based on publicly available population denominators. For example, in 15–19 year old women, test coverage estimates were from 3.1 per 100 (minimum) to 9.7 (maximum) in 2000, increasing to 39.3 in 2011, with corresponding diagnosis rates of 891.1 to 2488.6 in 2000 and 2786.6 in 2011^7^. In the baseline scenario of the model, we used the mid-points of the minimum and maximum estimates. As sensitivity analyses, we also ran the model on the minimum and maximum estimates only. Chlamydia testing data did not distinguish between tests provided to people with symptoms suggestive of infection with *C*. *trachomatis* and screening tests amongst people without symptoms.

### Chlamydia transmission model

**Figure 1:**
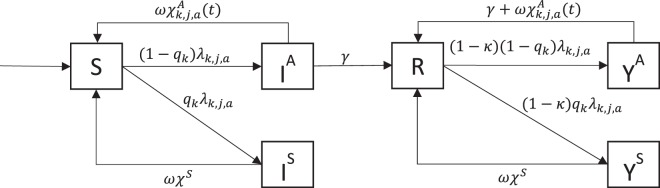
Schematic illustration of the *C. trachomatis* transmission model. S susceptible; I^A^ asymptomatically infected; I^S^ symptomatically infected; R partially immune; Y^A^ asymptomatic reinfection; Y^S^ symptomatic reinfection. Parameters are described in Table 1.

We developed a mathematical model to describe heterosexual *C*. *trachomatis* transmission in England from 2000 to 2011. The model uses differential equations and is described in detail in the Supplementary Information, part I; a brief summary is provided here. Model compartments and transmission rates are shown in Fig. 1 and Table 1.

**Table 1:** Description of parameters used in the transmission model and their prior distributions.

Parameter	Description	Unit	Prior distribution	Prior mean (95%CrI)	Ref.
*γ*	1/duration of untreated asymptomatic infection (1/years)	1/years	Normal (0.84, 0.02)	0.84 (0.80–0.88)	^17^
χ^*S*^	1/duration of the symptomatic period	1/years	Normal (11.1, 1.3)	11.1 (8.6–13.5)	^33^
*ω*	Probability of successful infection clearance after treatment	—	Normal (0.74, 0.02)	0.74 (0.71–0.78)	^43, 44^
*β*	Per partnership transmission probability	—	Uniform (0, 1)	0.5 (0.025–0.975)	
${\epsilon }$	Sexual mixing coefficient	—	Uniform (0, 1)	0.5 (0.025–0.975)	
*q*_*M*_	Fraction of symptomatic infections in men	—	Uniform (0, 1)	0.5 (0.025–0.975)	
*q*_*F*_	Fraction of symptomatic infections in women	—	Uniform (0, 1)	0.5 (0.025–0.975)	
*η*_1_	Parameter 1 for per capita ratio of screening between infected and susceptibles	—	Uniform (1, 10)	0.5 (0.025–0.975)	
log(*η*_2_)	Log transform of parameter 2 for per capita ratio of screening between infected and susceptibles	—	Uniform [−2, 1]	−0.5 (−1.925–0.925)	
*κ*	Factor by which susceptibility to subsequent infection is reduced through partial immunity	—	Uniform (0, 1)	0.5 (0.025–0.975)	

The model describes the transmission of chlamydia among susceptible people (*S*), infected people with a primary infection (*I*), people that have recovered naturally (*R*) and people with a repeated infection (*Y*). People are further stratified by sex *k* (men, women), age *a* (15–17, 18–19, 20–24, 25–34 and 35–44 years old) and sexual activity class *j* (low, high, defined by the average number of new heterosexual partners per year). Detailed age mixing behaviour and mixing between activity classes is modelled using methods proposed in a previous publication, where we accounted for differences in sexual behaviour reported by men and women^24^. People can switch between activity classes at a rate that is proportional to the size of these classes^25^. There is a time-dependent force of infection *λ*_*k,j,a*_ that involves the per partnership transmission probability (*β*) and the heterosexual mixing patterns between ages and sexual activity classes. A fraction *q* of all new infections is symptomatic, the remainder being asymptomatic. People in the recovered compartment are partially immune with a reduced susceptibility to reinfection.

We assumed that all symptomatically infected people receive a chlamydia test at rate χ^*S*^ and are subsequently treated. After treatment, symptomatically infected people become susceptible again at rate *ωχ*^*S*^, where *ω* is the probability of successfully clearing infection after treatment and 1/*χ*^*S*^ the average duration until treatment. Asymptomatically infected people can clear their infection naturally at rate *γ*, or are screened at a sex- and age-dependent rate ${\chi }_{k,a}^{A}(t)$ that depends on time *t*. The chlamydia screening rate amongst asymptomatic individuals differs according to the risk of being infected, which we refer to as differential screening coverage. In the model, we define differential screening coverage as the ratio of the screening rates in asymptomatic individuals who are infected with *C*. *trachomatis* and asymptomatic individuals who are uninfected. Arguably, this ratio decreases until 2011, as more screening is offered and becomes less targeted. We model differential screening coverage by a time-dependent parameter *η*_*k*,*a*_ (*t*) that decreases exponentially as a function of the total yearly number of screening tests ${{\Xi }}_{k,a}(t)$:

1 $${\eta }_{k,a}({{\Xi }}_{k,a})(t)=1+({\eta }_{1}-1)\exp (\,-\,{\eta }_{2}\,{{\Xi }}_{k,a}(t)))).$$

${\eta }_{k,a}({{\Xi }}_{k,a})$ starts at *η*_1_ and converges to one as the number of tests ${\Xi }_{k,a}$ becomes large, reflecting the situation in which screening is distributed homogeneously among all people. We then calculated the screening rate in asymptomatically infected people, ${\chi }_{k,a}^{A}(t)$, from ${{\Xi }}_{k,a}(t)$ and *η*_*k,a*_(*t*) by dividing the total number of screening tests in a given year by the total number of people eligible to receive screening, accounting for the differential screening coverage between asymptomatically infected and uninfected people:

2 $${\chi }_{k,a}^{A}(t)={\eta }_{k,a}\frac{{{\Xi }}_{k,a}(t)}{{\sum }_{j}[{S}_{k,j,a}(t)+{R}_{k,j,a}(t)+{\eta }_{k,a}\,({I}_{k,j,a}^{A}(t)+{Y}_{k,j,a}^{A}(t))]}.$$

### Parameter inference

We inferred the parameter values using Markov Chain Monte Carlo (MCMC) sampling (Table 1)^26^. We ran five separate MCMC chains for 10,000 MCMC steps. We assumed a binomial likelihood for the prevalence data and a negative binomial (NB) likelihood for the diagnoses data (Supplementary Information, part II). We checked convergence of the MCMC chains by computing the Gelman-Rubin convergence diagnostic (<1.1)^27^.

### Comparison of model variants

We used the chlamydia transmission model to investigate whether long-lasting partial immunity and differential screening coverage can explain discrepancies between model-predicted effects of screening and observed data in England. We considered four different model variants (Table 2). In models 1 and 2 we assumed no partial immunity (by fixing *κ* to zero), whereas models 3 and 4 can account for immunity (0 < *κ* ≤ 1, estimated using MCMC). In models 1 and 3, we assumed a differential screening coverage that does not change as screening increases in time, i.e. (*η*_1_ estimated using MCMC and fixing *η*_2_ to zero). In models 2 and 4 we allowed for a differential screening coverage that changes as screening increases (*η*_1_, e and *η*_2_ both estimated using MCMC). We compared the goodness of fit of the different model variants using the deviance information criterion (DIC)^28,29^. According to a rule of thumb, models with DIC within 1 or 2 points of the ‘best’ fitting model deserve consideration and models with DIC 3 or more points higher have considerably less support^29^.

**Table 2:** Summary of parameters (mean and 95% CrI of posterior distributions) for different models.

Parameter	Model 1: posterior mean (95%CrI)	Model 2: posterior mean (95%CrI)	Model 3: posterior mean (95%CrI)	Model 4: posterior mean (95%CrI)
*γ*	0.85 (0.82, 0.89)	0.86 (0.82, 0.9)	0.84 (0.8, 0.88)	0.84 (0.81, 0.88)
*χ* ^*S*^	11.23 (8.79, 13.55)	11.23 (8.94, 13.76)	10.95 (8.52, 13.84)	11 (8.37, 13.6)
*ω*	0.73 (0.7, 0.77)	0.73 (0.7, 0.77)	0.74 (0.71, 0.77)	0.74 (0.71, 0.77)
*β*	0.6 (0.56, 0.65)	0.59 (0.56, 0.64)	0.82 (0.7, 0.92)	0.8 (0.71, 0.92)
${\epsilon }$	0.76 (0.5, 0.95)	0.8 (0.58, 0.97)	0.8 (0.58, 0.96)	0.84 (0.63, 0.97)
*q*_*M*_	0.14 (0.09, 0.2)	0.1 (0.04, 0.17)	0.15 (0.1, 0.2)	0.1 (0.04, 0.17)
*q*_*F*_	0.16 (0.07, 0.25)	0.11 (0.02, 0.21)	0.17 (0.09, 0.26)	0.11 (0.02, 0.23)
*η*_1_	1.99 (1.18, 2.84)	5.14 (2.08, 8.95)	2.29 (1.47, 3.26)	5.32 (2.24, 9.05)
*η*_2_	0*	1.06 (0.54, 1.62)	0*	0.93 (0.55, 1.30)
*κ*	0*	0*	0.69 (0.45, 0.89)	0.68 (0.46, 0.87)
Log likelihood	−705.45 (−709.76, −702.33)	−702.13 (−707.62, −698.42)	−692.63 (−697.86, −689.09)	−689.96 (−695.26, −686.49)
DIC	1421	1420	1397	1388

In these models we used the midpoints of minimum and maximum estimates for tests and diagnoses from Chandra and colleagues^7^. For parameter inference. The last two rows show the fit statistics of the models. *Kept as fixed values in these models.

Model code was implemented using R version 3.4.0 and can be obtained from https://github.com/joostsmid/ct-screening.

## Results

The posterior probability distributions for the model-estimated parameters were different for each model variant and resulted in a different goodness of fit (DIC) to the empirical data on *C*. *trachomatis* prevalence and diagnoses (Table 2). Comparing the DIC values allowed us to investigate the validity of the assumptions distinguishing the four model variants. A model including a decreasing per capita ratio of screening tests in infected compared to susceptible people and partial immunity (model 4) described the data better (lowest DIC) than models where the composition of the screened population was assumed fixed in time or without partial immunity (Table 2). A model including partial immunity but no changes over time in the composition of the screened population (model 3) fitted second best to the data with a 9 points’ higher DIC value when using the midpoints of the minimum and maximum estimates for tests and diagnoses. In a sensitivity analysis, using the minimum and maximum estimates, model 3 and 4 fitted equally well when using minimum estimates. When using maximum estimates, model 3 fitted best. In all scenarios, models without partial immunity fitted the data considerably worse (Supplementary Information, part III). Hence, our results with respect to partial immunity are robust to different assumptions about the data on tests and diagnoses. The evidence for a decrease in the proportion of tests done in chlamydia-infected individuals is less conclusive. Further results focus on the baseline scenario, with models fitted to the midpoint estimates.

Partial immunity decreased susceptibility to reinfection after natural clearance of a first infection by 68% (95% Bayesian credible interval (CrI) 46–87%) in model 4 (Table 2). The estimate of partial immunity in the second-best fitting model 3 (fixed per capita ratio of tests in infected compared to susceptible people) was similar (69%, 95% CrI 45–89%). When using minimum or maximum estimates for tests and diagnoses, estimates of partial immunity were also similar (Supplementary Information, part III).

**Figure 2:**
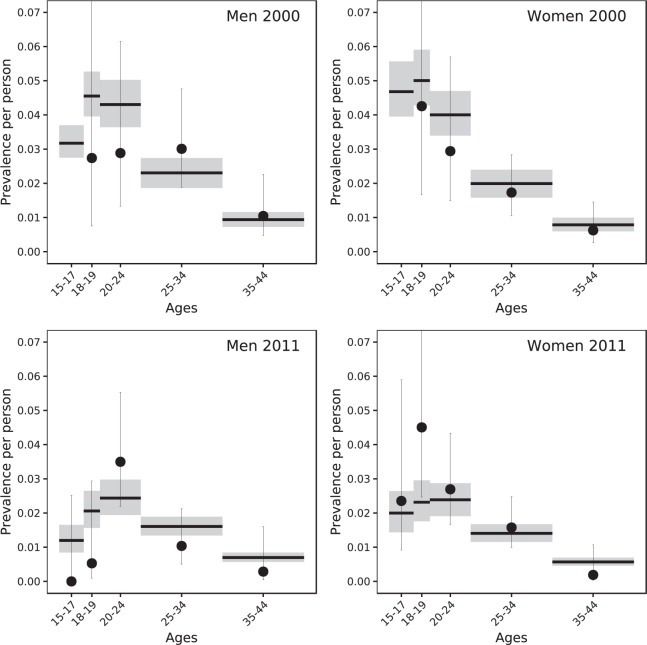
Fit of model 3 (full model) to age-specific chlamydia prevalence for men and women in 2000 and 2011. Grey boxes and horizontal lines: posterior mean and 95% Bayesian credible intervals. Black dots and vertical bars: Estimated prevalence from Natsal-2 (2000) and Natsal-3 (2011) (mean and 95% confidence intervals).

Figure 2 shows the posterior prevalence predicted by the best-fitting model 4 and the observed levels of *C*. *trachomatis* prevalence in England in 1999–2000 and 2010–2011. The uncertainty about the prevalence estimates from empirical data is large due to small sample sizes^8,9^. The model predicted a relative drop in prevalence for women between 15–24 years of 50% (95% CrI 44–54%) and for men between 15–24 years of 48% (95% CrI 44–52%), also see Fig. 5. The mean model-predicted prevalence falls within the confidence intervals of the prevalence data for all age groups and both periods, with the exception of women aged 18–19 years in 2011. However, some inconsistencies remain between the observed and model-predicted chlamydia prevalence. In particular, for women the mean model-predicted prevalence is higher than the mean prevalence from data for 2000 and lower for 2011 for almost all age groups.

**Figure 3:**
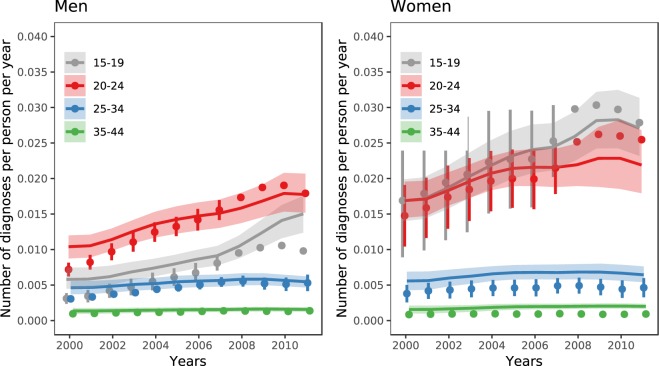
Fit of model 4 (full model) to age-specific per capita number of diagnoses for men and women between 2000 and 2011. Coloured lines and shaded areas: posterior mean and 95% Bayesian credible intervals. Vertical bars and dots: Minimum and maximum estimates for number of diagnoses from Chandra and colleagues^7^, and midpoints of these estimates (used for fitting).

Empirical data show that the per capita number of diagnoses of *C*. *trachomatis* infections has increased between 2000 and 2011 (Fig. 3). Although model 4 could roughly reproduce this trend, some differences remain between the model fit and the data. In general, the model shows a less steep increase in the number of diagnoses from 2000 to 2011 for men and women between 20–24 years than was estimated by Chandra and colleagues^7^. For men aged 15–19 years, the model-predicted number of diagnoses increased more steeply than observed from data. Model fits for models 1–3 are shown in the Supplementary Information, part IV. Similarly, model 4 reproduced the trend in declining positivity rates of chlamydia tests over time (Fig. S12, Supplementary Information part V). There were some differences between model-predicted and observed positivity rates, particularly in men.

**Figure 4:**
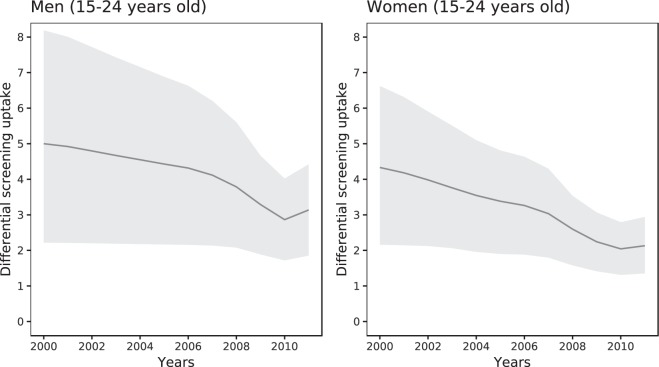
Differential screening coverage: Model-estimated change in the ratio of the screening rates in infected vs. susceptible individuals for men and women of 16–24 years old between 2000 and 2011. Coloured lines and shaded areas: Posterior mean and 95% Bayesian credible intervals.

**Figure 5:**
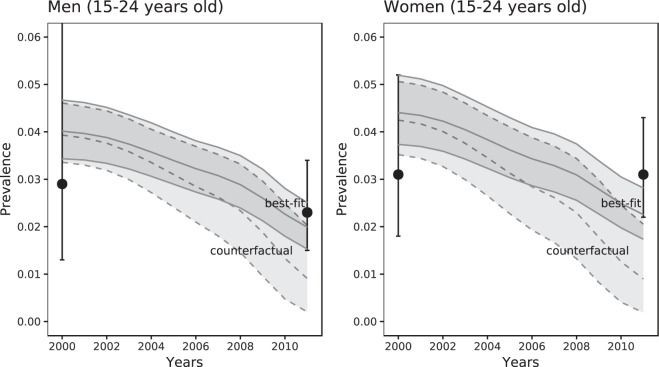
Model-estimated change in chlamydia prevalence in men and women between 2000 and 2011. Best-fit: Full model (model 4) including changes in the proportion of tests done in infected individuals and partial immunity (posterior mean and 95% Bayesian credible intervals). Hypothetical: Scenario in which the proportion of screening tests in infected individuals is kept at the same level as was estimated for 2000. Error bars for 2000: chlamydia prevalence in men/women aged 18–24 from Natsal-2; error bars for 2011: chlamydia prevalence in men/women aged 16–24 from Natsal-3 (mean and 95% confidence intervals).

In model 4, differential screening coverage decreased in time (Fig. 4) due to increased testing and concurrent with increasing overall screening rates and screening rates in infected people (Figs S7–S9, Supplementary Information part V). The expected screening rate was in the full model 4.3 (95% CrI 2.2–6.6) times higher for infected than for uninfected women between 15–24 years in 2000; this decreased to 2.1 (95% CrI 1.4–2.9) in 2011. For men, this ratio decreased from 5.0 (95% CrI 2.2–8.2) in 2000 to 3.1 (95% CrI 1.9–4.4) in 2011 (Fig. 4). The Bayesian credible intervals around these estimates are large. For men and women aged 25–44 years, we found no decrease in the ratio. The decrease in differential screening coverage in the model is the consequence of increased testing volume and not of decreased chlamydia prevalence, but it can affect prevalence. To illustrate this, we considered the counterfactual scenario where differential screening coverage remained at the level from 2000, as estimated in model 4. We used the estimated posterior distribution for *η*_1_ and fixed *η*_2_ to zero. Simulating this scenario resulted in considerably greater reductions in chlamydia prevalence than with model 4, in which differential screening coverage was allowed to change (Fig. 5).

## Discussion

In this study, we compared four model variants in a compartmental model of *C*. *trachomatis* infection dynamics that includes partial immunity after natural clearance of infection and changes in differential screening coverage over time. In our baseline scenario with regard to the numbers of chlamydia tests and diagnoses, the model variant that fitted best to empirical data about *C*. *trachomatis* prevalence and diagnoses in England between 2000 and 2011 (model 4) included both partial immunity (a reduction of susceptibility of 46–87%) and a decreasing proportion of screening tests done in infected people over time. Although both factors attenuate the model-predicted effectiveness of screening, the model including both factors still predicted a decrease of chlamydia prevalence between 2000 and 2011 of 45–55% in the age groups targeted for screening.

A strength of this study is the availability of time-series data about chlamydia testing and diagnosis data^7^, and repeated cross-sectional surveys of *C*. *trachomatis* prevalence and sexual behavioural data from the same population over the same time period^22,23^. The testing data avoided the need, in other modelling studies, for strong assumptions about (unobserved) levels of screening coverage^11,13,30^. Second, because of the importance of age as a risk factor for chlamydia, we included detailed age structure and age-dependent sexual behaviour in our model, which allowed us to estimate chlamydia prevalence in different age groups and fitted these to data about age-specific prevalence and diagnoses^24^. We made optimal use of age-specific data to estimate the values about the unknown model parameters, including those quantifying immunity and the distribution of screening tests. Third, by synthesising the data in a Bayesian framework we could quantify the distinct effects of partial immunity and dynamic changes in the distribution of screening tests, whilst accounting for uncertainty about the model parameters.

Our study has also limitations. First, the testing and diagnosis data, rather than the chlamydia prevalence data mainly drove the Bayesian inference in our model. The statistical uncertainty of the prevalence data was higher than that of the diagnoses data, because the Natsal surveys included a small sample of the target population for two periods only (1999–2001 and 2010–2012), whereas the data about diagnoses are from the whole of England for each year between 2000–2011. Moreover, the prevalence data are probably reasonably accurate because they are from a well- characterised population, corrected for representativeness using post-stratification weights, but imprecise. The testing and diagnoses data are less accurate in the earlier years but very precise. To account for the lower accuracy of the diagnosis data, we modelled their likelihood using a negative binomial distribution instead of a Poisson distribution, which introduced a moderate dispersion in the diagnoses data. Second, the assumption of an exponential relationship between the total number of tests *T* in a given year and the per capita ratio of testing in infected compared with susceptible people *η*(*T*) in that year might be an over-simplification of geographical and temporal changes in screening^31^. The phased geographical roll-out of the NCSP probably accounted for most of the increase in testing from 2003–2007. After nationwide implementation, when targets for screening coverage were introduced, an increasing proportion of tests was probably done in those at lower risk^32^. Then, an exponential function makes sense because convergence to one as the number of tests becomes large indicates the increasingly equal distribution of tests among all sexually active people. Third, we had to be selective about the parameters included in the model because the MCMC algorithm would otherwise not converge. We could, therefore, not consider all heterogeneity between individuals in susceptibility and biological response to infection. We assumed only one level of partial immunity, which is a likely over-simplification, given the possibility of heterogeneity in immune response at the level of the individual or strain-specific immunity, and potential effects of repeat infection. We also did not consider waning immunity. Further, we assumed that the transmission probabilities and duration of untreated asymptomatic infection were the same for men and women. There is much uncertainty about these estimates but our assumptions are in line with those used in other modelling studies^33,34^. It is unclear how these assumptions and levels of heterogeneity would affect our results. Finally, we assumed the sexual behaviour parameters to be fixed and did not consider changes in sexual behaviour between 2000–2011. Although there were only minor differences in sexual behaviour data between Natsal-2 and Natsal-3^22^, we acknowledge that this is assumption may have caused discrepancies between the true and the model-predicted prevalence and incidence over time.

Mathematical modelling studies of *C*. *trachomatis* often over-estimate the effects of screening interventions on prevalence considerably when compared with empirical estimates in repeated cross-sectional studies. The results of our study suggest that including protective immunity after natural clearance of *C*. *trachomatis*^10–14^ and changes over time in the distribution of screening tests among modelled subpopulations helps to better account for the marginal change in chlamydia prevalence estimated in Natsal-2 and Natsal-3. However, our model did not fully reconcile the model predictions with the data either, with a model-predicted decrease of *C*. *trachomatis* prevalence between 2000–2011 that is still somewhat larger than expected from data^9^. According to our model, screening and treating *C*. *trachomatis-*infected people resulted in a reduction in prevalence even when accounting for immunity. This finding does not support the results of a modelling study that suggested that widespread testing and treatment diminish population level immunity and can result in an increase in incidence and prevalence^35^. Other factors have been proposed as reasons for the similarity of estimated *C*. *trachomatis* prevalence in the Natsal-2 and Natsal-3 surveys. First, increases in sexual risk-taking behaviour could have resulted in increased transmission of *C*. *trachomatis*, countering the effects of screening. However, amongst the sexual behaviours measured in Natsal-2 and Natsal-3, the only difference among heterosexual adults was a slight decrease in the proportion of men with multiple partners with whom no condoms was used^22^. We do not believe that this change would be sufficient to abolish the model-predicted reduction in prevalence and we did not investigate this possibility because our model did not explicitly include condom use. Second, a temporary reduction in *C*. *trachomatis* prevalence could have occurred during the period between the Natsal surveys. In the modelling study by Lewis and White, which also used the data on chlamydia testing and diagnoses reported by Chandra and colleagues, model-inferred prevalence decreased until 2008 and then increased back to baseline, attributed to a decrease in testing by the authors^15^. That model did not take into account differential screening coverage over time and inferred large increases in incidence to balance the increase in screening rates^36^. In theory, a reduction in the rate of successful partner notification could also limit the impact of a chlamydia screening intervention. However, in a study that compared a pair model including partner notification with a simpler model without partnerships^37^, changes in partner notification rates resulted in very modest changes in incidence. Third, it has been suggested that *C*. *trachomatis* transmission is maintained by infection in the female rectum that is not adequately treated^38,39^. We deemed an analysis of the role of anorectal infections beyond the scope of this study because of the uncertainty about autoinoculation probabilities.

Our findings have implications for future research and for public health. Mathematical modelling often provides the first indications about the potential effects of interventions to control infectious diseases. Arguably, data from population-based studies, preferably from randomised controlled trials, should be obtained to inform further modelling of future impact before the introduction of a screening programme. For *C*. *trachomatis*, chlamydia testing became widespread before either modelling or trials had been conducted. Public health decision-makers and researchers should jointly determine requirements for monitoring and epidemiological, clinical and basic research that will address gaps in the evidence. Clinical immunological studies that can provide more information about the strength and duration of immunity following natural clearance of *C*. *trachomatis* would be very valuable. Definitive answers will have to await the outcomes of ongoing research for better validated serological markers of immunity than we have at present^40^. Our model predicted a larger reduction in *C*. *trachomatis* prevalence if the ratio of screening coverage in infected compared with uninfected individuals remained high. This finding has implications for defining targets for screening performance. After 2011 in the NCSP in England, targets for screening coverage were replaced by indicators for diagnosis rates to maintain high levels of diagnosed infections for a given test volume^41^. This resulted in a decreased testing volume but an increased percentage testing positive. The optimal diagnosis rate and its relationship with population prevalence, however, remains unknown^42^, so further data about the relationship between sexual behaviour, chlamydia testing and its outcomes in different subpopulations over time are needed.

In conclusion, the results from our baseline scenario suggest that partial immunity against reinfection and changes in differential screening coverage over time might have limited the reduction in *C*. *trachomatis* prevalence that would be expected for the level of screening coverage achieved in England. The full range of factors that account for discrepancies between the observed data and model-predictions of changes in *C*. *trachomatis* prevalence has not been elucidated. To reduce the gap between modelling and data, advances are needed in knowledge about factors influencing the coverage of chlamydia screening, the immunology of *C*. *trachomatis* and changes in *C*. *trachomatis* prevalence at the population level.

## Supplementary information

Supplementary Information
