# Supplementary material for: Discrepancies between observed data and predictions from mathematical modelling of the impact of screening interventions on Chlamydia trachomatis prevalence
Source: Sci Rep. 2019 May 17;9:7547. doi: 10.1038/s41598-019-44003-x (PMC6525258; doi:10.1038/s41598-019-44003-x)
Supplement: Supplementary file 1 — Supplementary Information [file 41598_2019_44003_MOESM1_ESM.pdf]

Supplementary Information

Discrepancies between observed data and predictions from mathematical modelling of the impact of screening interventions on *Chlamydia trachomatis* prevalence

Joost Smid[a], Christian L. Althaus*[a] Nicola Low[a]

[a]University of Bern, Institute of Social and Preventive Medicine (ISPM), Switzerland

*Corresponding author: Phone: +41-316315697; E-mail: christian.althaus@ispm.unibe.ch; Address: Mittelstrasse 43, 3012 Bern, Switzerland

Contents

Part I: *Chlamydia trachomatis* transmission model 3
Overview of the model 3
Becoming sexually active 4
Becoming infected by C. trachomatis 5
Screening and treatment for chlamydia infection 6
Natural recovery and immunity 7
Part II: Model parameterisation 7
Sexual behaviour 7
Chlamydia tests and diagnoses 7
Parameters inferred using Markov Chain Monte Carlo (MCMC) sampling 8
Part III: Posterior distribution and model fit when using minimum and maximum estimates 11
Part IV: Model fits for model 1-3 12
Part V: Additional figures 18
References 22

Figures

Figure S1: Fit of model 1 (excluding partial immunity and changes in the proportion of tests done in infected individuals) to age-specific chlamydia prevalence for men and women in 2000 and 2011. Grey boxes and horizontal lines: posterior mean and 95% Bayesian credible intervals. Black dots and vertical bars: Estimated prevalence from Natsal-2 (2000) and Natsal-3 (2011) (mean and 95% confidence intervals). 13
Figure S2: Fit of model 1 (excluding partial immunity and changes in the proportion of tests done in infected individuals) to age-specific per capita number of diagnoses for men and women between 2000 and 2011. Coloured lines and shaded areas: posterior mean and 95% Bayesian

credible intervals. Vertical bars and dots: Minimum and maximum estimates for number of diagnoses from Chandra et al, and midpoints of these estimates (used for fitting). 14
Figure S3: Fit of model 2 (including changes in the proportion of tests done in infected individuals; excluding partial immunity) to age-specific chlamydia prevalence for men and women in 2000 and 2011. Grey boxes and horizontal lines: posterior mean and 95% Bayesian credible intervals. Black dots and vertical bars: Estimated prevalence from Natsal-2 (2000) and Natsal-3 (2011) (mean and 95% confidence intervals). 15
Figure S4: Fit of model 2 (including changes in the proportion of tests done in infected individuals; excluding partial immunity) to age-specific per capita number of diagnoses for men and women between 2000 and 2011. Coloured lines and shaded areas: posterior mean and 95% Bayesian credible intervals. Vertical bars and dots: Minimum and maximum estimates for number of diagnoses from Chandra et al, and midpoints of these estimates (used for fitting). 16
Figure S5: Fit of model 3 (including partial immunity; excluding changes in the proportion of tests done in infected individuals) to age-specific chlamydia prevalence for men and women in 2000 and 2011. Grey boxes and horizontal lines: posterior mean and 95% Bayesian credible intervals. Black dots and vertical bars: Estimated prevalence from Natsal-2 (2000) and Natsal-3 (2011) (mean and 95% confidence intervals). 17
Figure S6: Fit of model 3 (including partial immunity; excluding changes in the proportion of tests done in infected individuals) to age-specific per capita number of diagnoses for men and women between 2000 and 2011. Coloured lines and shaded areas: posterior mean and 95% Bayesian credible intervals. Vertical bars and dots: Minimum and maximum estimates for number of diagnoses from Chandra et al, and midpoints of these estimates (used for fitting). 18
Figure S7: Testing rates per person per year from Chandra et al. The end points of the vertical bars represent the minimum and maximum estimates for number of tests between 2000 and 2011. The dots represent the midpoints of the minimum and maximum estimates, used in the model. 19
Figure S8: Model-estimated screening rates in asymptomatically infected men and women in different age groups between 2000 and 2011. Coloured lines and shaded areas: posterior mean and 95% Bayesian credible intervals 19
Figure S9: Model-estimated number of screening tests per person per year in all men and women in different age groups between 2000 and 2011. Coloured lines and shaded areas: posterior mean and 95% Bayesian credible intervals 20
Figure S10: Model-estimated prevalence in men and women in different age groups between 2000 and 2011. Coloured lines and shaded areas: posterior mean and 95% Bayesian credible intervals 20
Figure S11: Model-estimated incidence rates per person per year in men and women in different age groups between 2000 and 2011. Coloured lines and shaded areas: posterior mean and 95% Bayesian credible intervals 21
Figure S12: Positivity rates of chlamydia tests from Chandra et al, and model-estimates positivity rates for men and women in different age groups between 2000 and 2011. Coloured lines and shaded areas: posterior mean and 95% Bayesian credible intervals. Vertical bars and dots: Minimum and maximum estimates from data, and midpoints of these estimates. Positivity rates were computes by dividing the number of diagnoses in a sex and age stratum by the number of tests done in that stratum. 21

Supplementary Information

Part I: *Chlamydia trachomatis* transmission model

Overview of the model

Our transmission model for chlamydia has two sex compartments, five age compartments (15-17, 18-19, 20-24, 25-34 and 35-44), two compartments for heterogeneity in sexual risk behaviour (low and high, defined by the average number of new heterosexual partners per year) and seven infection compartments (people not having had sex yet, U; susceptible, S; asymptomatically infected after a first infection, I^A; symptomatically infected after a first infection, I^S; recovered from a previous infection, R; asymptomatically infected after a repeat infection, Y^A; and symptomatically infected after a repeat infection, Y^S). The model describes the flow of densities of persons through these compartments by ordinary differential equations (ODE). The model dynamics are described by the following set of equations:

$$\frac{dU_{k,a}}{dt} = \alpha p_{k,a} U_{k,a-1} - \alpha\, U_{k,a} \qquad (1)$$

$$\frac{dS_{k,j,a}}{dt} = f_{k,j}\alpha(1-p_{k,a})U_{k,a-1} + \alpha S_{k,j,a-1} - \alpha S_{k,j,a} - \lambda_{k,j,a}S_{k,j,a} + \omega\, \chi^A_{k,a} I^A_{k,j,a} + \omega\chi^S\, I^S_{k,j,a} + m\, f_{k,j} \sum_i S_{k,i,a} - m\, S_{k,j,a} \qquad (2)$$

$$\frac{dI^A_{k,j,a}}{dt} = \alpha\, I^A_{k,j,a-1} - \alpha I^A_{k,j,a} + (1-q_k)\, \lambda_{k,j,a}S_{k,j,a} - \left(\gamma + \omega\, \chi^A_{k,a}\right)I^A_{k,j,a} + m\, f_{k,j} \sum_i I^A_{k,i,a} - m\, I^A_{k,j,a} \qquad (3)$$

$$\frac{dI^S_{k,j,a}}{dt} = \alpha\, I^S_{k,j,a-1} - \alpha I^S_{k,j,a} + q_k\, \lambda_{k,j,a}S_{k,j,a} - \omega\, \chi^S\, I^S_{k,j,a} + m\, f_{k,j} \sum_i I^S_{k,i,a} - m\, I^S_{k,j,a} \qquad (4)$$

$$\frac{dR_{k,j,a}}{dt} = \alpha\, R_{k,j,a-1} - \alpha R_{k,j,a} + \gamma\, (I^A_{k,j,a} + Y^A_{k,j,a}) - (1-\kappa)\lambda_{k,j,a}\, R_{k,j,a} + \left(\omega\, \chi^A_{k,a} Y^A_{k,j,a} + \omega\chi^S\, Y^S_{k,j,a}\right) + m\, f_{k,j} \sum_i R_{k,i,a} - m\, R_{k,j,a} \qquad (5)$$

$$\frac{dY^A_{k,j,a}}{dt} = \alpha\, Y^A_{k,j,a-1} - \alpha Y^A_{k,j,a} + (1-q_k)\,(1-\kappa)\lambda_{k,j,a}R_{k,j,a} - \left(\gamma + \omega\, \chi^A_{k,a}\right)Y^A_{k,j,a} + m\, f_{k,j} \sum_i Y^A_{k,i,a} - m\, Y^A_{k,j,a} \qquad (6)$$

$$\frac{dY^S_{k,j,a}}{dt} = \alpha\, Y^S_{k,j,a-1} - \alpha Y^S_{k,j,a} + q_k(1-\kappa)\, \lambda_{k,j,a}R_{k,j,a} - \omega\, \chi^S\, Y^S_{k,j,a} + m\, f_{k,j} \sum_i Y^S_{k,i,a} - m\, Y^S_{k,j,a} \qquad (7)$$

Supplementary Information

Ageing

Ageing, at rate α, moves persons from one age group to the next. The depletion of persons who age beyond $a = a_{max}$, where a_{max} denotes the largest age group considered,

is compensated by the equivalent emergence b_k of persons in the youngest age class so that the total population size remains constant:

$$b_k = \alpha\,(U_{k,a_{max}} + \sum_j (S_{k,j,a_{max}} + I^A_{k,j,a_{max}} + I^S_{k,j,a_{max}} + R_{k,j,a_{max}} + Y^A_{k,j,a_{max}} + Y^S_{k,j,a_{max}})) \tag{8}$$

Becoming sexually active

In the youngest considered age group a_{min}, the compartment of persons not having had sex ($U_{k,a_{min}}$) is populated at a rate $\alpha p_{k,a_{min}} b_k$, where $p_{k,a_{min}}$ is the probability that an individual of gender k has not had sex before age a_{min}. The compartment of susceptibles $S_{k,j,a_{min}}$ is populated at rate $\alpha(1 - p_{k,a_{min}}) f_{k,j} b_k$, where $f_{k,j}$ represents the probability of moving to the low ($j = 1$) or high ($j = 2$) sexual activity class. For $a > a_{min}$, the inflow of persons into $U_{k,a}$ stems from the aging of persons not having had sex from the preceding younger age group. This occurs at a rate $\alpha\, p_{k,a}\, U_{k,a-1}$, where $p_{k,a}$ represents the conditional probability for a persons that has not had sex before age $a - 1$ to also have not had sex until the end of age $a - 1$. Alternatively, persons having sex for the first time at age $a - 1$ (i.e. becoming sexually active) move to the $S_{k,j,a}$ compartment at a rate $\alpha\left(1 - p_{k,a}\right) f_{k,j} U_{k,a-1}$. Persons switch between sexual activity classes after every year at rate m .[1]

Becoming infected by *C. trachomatis*

Susceptible persons can be infected by infectious persons at rate $\lambda_{k,j,a}$, the force of infection:

$$\lambda_{k,j,a} = \beta \sum_{j'=1}^{2} \sum_{a'} c_{k,j,j',a,a'} \, \rho_{k,j,j',a,a'} \frac{I^A_{k',j',a'} + I^S_{k',j',a'}(t) + Y^A_{k',j',a'} + Y^S_{k',j',a'}(t)}{N_{k',j',a'}(t)} \tag{9}$$

The quantity $N_{k,j,a}$ represents the total number of persons in class (k, j, a) that is sexually active:

$$N_{k,j,a} = S_{k,j,a} + I^A_{k,j,a} + I^S_{k,j,a} + R_{k,j,a} + Y^A_{k,j,a} + Y^S_{k,j,a} \tag{10}$$

β is the per partnership transmission probability. $\rho_{k,j,j',a,a'}$ represents the probability of a sexually active person in class (k, j, a) having a new sexual partnership with a partner in class (k', j', a'). It is calculated assuming that mixing between activity classes is in between assortative (where the delta function $\delta_{j,j'} = 1$) and proportionate ($\delta_{j,j'} = 0$), using the assortativity constant $\epsilon \in [0,1]$: [2]

$$\rho_{k,j,j',a,a'} = \rho_{k,a,a'} \left(\epsilon \, \delta_{j,j'} + (1 - \epsilon) \frac{N_{k',j',a'} c_{k',j',a'}}{\sum_{i=1}^{n_J} N_{k',i,a'} c_{k',i,a'}} \right) \tag{11}$$

Here $\rho_{k,a,a'}$ is the probability for a sexually active person in class (k, a) to have a new heterosexual partner of age a'. The mean partner change rates, defined as the mean number of new heterosexual partners per year for persons in class (k, j, a), are represented

by $c_{k,j,a}$. Variables $c_{k,j,j',a,a'}$ in equation (9) represent the adjusted partner-change rates, balancing data discrepancies about partner change rates between men and women. We follow the approach of Garnett et al.[2] by considering the ratio of new sexual partners per year reported by men (numerator) and women (denominator) for each age and activity class:

$$B_{j,j',a,a'} = \frac{N_{k,j,a} c_{k,j,a} \rho_{k,j,j',a,a'}}{N_{k',j',a'} c_{k',j',a'} \rho_{k',j',j,a',a}} \tag{12}$$

An adjusted set of change rates of sexual partners provides the necessary balancing:[2]

$$c_{k,j,j',a,a'} = c_{k,j,a} B_{j,j',a,a'}^{0.5} \tag{13}$$

$$c_{k',j',j,a',a} = c_{k',j',a'} B_{j,j',a,a'}^{0.5}$$

A fraction $(1 - q_k)$ of persons that become infected moves to the I^A or Y^A compartment (depending on whether it is the first infection or not); the remaining fraction q_k moving to the I^S or Y^S compartment.

Screening and treatment for chlamydia infection

Symptomatically infected persons are treated at rate χ^S. Asymptomatically infected persons are screened at rate $\chi_{k,a}^A(t)$, which is time-dependent, depending on the total number of screening tests provided and differential screening coverage at time t (see main text). Infected persons, who are tested and found positive, remain infected if treatment fails. If successfully treated people are reinfected by an infected partner, then they become chlamydia positive again soon after their treatment. We modelled this by reducing the treatment rate by $\omega = \omega_1 \omega_2$, where ω_1 is the probability that a treatment is successful and

ω_2 is the probability of not being immediately reinfected by a partner after treatment. Thus, we implicitly assume that persons that were (a)symptomatically infected during the first episode of infection, will again be (a)symptomatically infected after re-infection.

Natural recovery and immunity

Asymptomatically infected people can also recover naturally from infection at rate γ. We assume that only persons who clear *C. trachomatis* naturally become partially immune, which moves them to the R compartment. In the model, this means that they have a reduced probability to be infected again. The force of infection, $\lambda_{k,j,a}$ is for them reduced by a factor $(1-\kappa)$.

Part II: Model parameterisation

Sexual behaviour

The sexual behaviour parameters $c_{k,j,a}$, $\rho_{k,a,a'}$, $p_{k,a}$ and $f_{k,j}$ were obtained from the second and third British National Surveys of Sexual Attitudes and Lifestyles (Natsal-2 and Natsal-3)[3,4] using data about the number of new heterosexual partners in the last year, the respondent's age at first heterosexual intercourse and the respondent's age and partner ages at the time of first sexual intercourse with the first, second and third most recent heterosexual partner. We used approaches to parameterise the data as described previously.[5] Using these approaches, parameters that differ by age class were smoothed across age classes.[5]

Chlamydia tests and diagnoses

C. trachomatis testing data collated by Public Health England were obtained from a publication by Chandra et al.[6] They provide minimum and maximum estimates for the per-capita number of chlamydia tests $\tau_{k,a,y}^{min}$ and $\tau_{k,a,y}^{max}$, done in men and women of different age groups (15-19, 20-24, 25-34 and 35-45) in England in the years $y \in \{2000, 2001, \ldots, 2011\}$.

The estimates include tests screening tests amongst asymptomatic people and tests done because of symptomatic infection. The model presented in the main text of this article, uses the mean values between the minimum and maximum estimates:

$$\tau_{k,a,y} := (\tau_{k,a,y}^{min} + \tau_{k,a,y}^{max})/2 \tag{14}$$

In part IV of the Supplementary Information, we present model results when using the minimum and maximum estimates of tests and diagnoses separately. We assumed that before 1990 no screening tests were done (only symptomatically infected persons were treated) and that screening increased linearly to the screening coverage as of 2000 in the period 1990-2000. We define $\Xi_{k,a}(y)$ as the total number of screening tests per year for asymptomatic infections, provided to all persons in (k, a) in year y. Using data on the total number of chlamydia tests in years 2000-2011 ($\tau_{k,a,y}$), we model $\Xi_{k,a}(y)$ as:

$$\Xi_{k,a}(y) = \begin{cases} 0 & y < 1990 \\ \frac{1}{10}(y - 1990)\left[\tau_{k,a,2000} - \sum_j I_{k,j,a}^S(y)\,\chi^S\right] & 1990 \le y < 2000 \\ \tau_{k,a,y} - \sum_j I_{k,j,a}^S(y)\,\chi^S & 2000 \le y < 2012 \\ \tau_{k,a,2012} - \sum_j I_{k,j,a}^S(y)\,\chi^S & y \ge 2012 \end{cases} \tag{15}$$

We used equations (1) and (2) in the main text to compute the per person screening rates from $\Xi_{k,a}(y)$.

Parameters inferred using Markov Chain Monte Carlo (MCMC) sampling

Values for model-specific parameters (β and ϵ) and uncertain or unknown parameters ($q_k, \eta_1, \eta_2, \kappa$) were inferred using MCMC sampling using Metropolis Hasting algorithm [7]. For every MCMC simulation, values were drawn from prior distributions of these parameters

and used to run the model into a steady state. Subsequently, their values were resampled according to a rejection criterion through which the total likelihood of the model was explored, using data about age- and sex-specific chlamydia positivity (prevalence data), and about age- and sex-specific number of diagnoses in the years 2000-2011 (diagnosis data).

We use chlamydia prevalence data from Natsal-2 and Natsal-3. We used the survey weights to compute the expected number of chlamydia infected people in a certain stratum of sex and age ($x_{k,a}$) and the total number of people in that stratum ($X_{k,a}$). Because of the weights, this resulted in numbers with fractions. We rounded these numbers to integers to use them in binomial likelihoods, acknowledging that this is an approximation of the true uncertainty about chlamydia prevalence within an sex/age stratum:

$$\mathcal{L}\left(x_{k,a} \middle| M_{k,a}(\theta), X_{k,a}\right) \quad = Binom(x_{k,a}|M_{k,a}(\theta), X_{k,a}) \tag{16}$$

where θ is the vector of parameter values, $M_{k,a}(\theta) = \frac{\sum_j I^A_{k,j,a}+I^S_{k,j,a}+Y^A_{k,j,a}+Y^S_{k,j,a}}{\sum_j N_{k,j,a}}(\theta)$ is the model-computed prevalence.

We simulate the number of diagnoses in the model by adding an additional compartment D (number of diagnoses) to the model. It is computed dynamically as

$$\frac{dD_{k,j,a}}{dt} = \chi^A_{k,a}(t)\ (I^A_{k,j,a} + Y^A_{k,j,a}) + \chi^S(I^S_{k,j,a} + Y^S_{k,j,a}) \tag{17}$$

To calculate the yearly number of new diagnoses $D_{k,j,a,y}$, we subtract the diagnoses in previous years from the total number of diagnoses after simulation of each year. These are compared to the data about number of diagnoses from Chandra *et al* [6]. We again use the

midpoints between minimum and maximum estimates of the number of diagnoses ($d_{k,a,y}$). We assume that these data are observations from a negative binomial likelihood:

$$\mathcal{L}\left(d_{k,a,y} = \frac{D_{k,a,y}^{min} + D_{k,a,y}^{max}}{2}\right) \tag{18}$$

$$= NegBin(\mu = D_{k,a,y}(\theta), var = D_{k,a,y}(\theta) * f_v)$$

where θ is the vector of parameter values, $D_{k,a,y}(\theta) = \sum_j D_{k,j,a,y}(\theta)$ is the model-computed number of diagnoses, $D_{k,a,y}^{min}$ and $D_{k,a,y}^{max}$are the minimum and maximum estimates for the number of diagnoses in sex k and age group a in year y, and f_v is a factor by which the model-computed number of diagnoses should be multiplied to get the variance of the negative binomial distribution, representing its dispersion. This parameterisation $NegBin(\mu, var)$ is sometimes referred to as the "ecological parameterisation" of the negative binomial distribution,[8] p. 165. In our case, the expected values are $D_{k,a,y}(\theta)$ but it has a more dispersed distribution as the Poisson distribution through the factor $f_v \geq 1$. If $f_v = 1$ then this distribution is equal to the Poisson distribution. The relation with the original parametrization of the negative binomial distribution ($NegBin(r, p)$) is $r = \frac{\mu^2}{var-\mu}$ and $p = \frac{var-\mu}{var}$. So we take

$$var = \frac{M_{k,a}(\theta)^2}{M_{k,a}(\theta)*f_v - M_{k,a}(\theta)} = \frac{M_{k,a}(\theta)}{f_v - 1} \tag{19}$$

The dispersion parameter f_v is not inferred in the MCMC sampling algorithm but, instead, a value of 200 is assumed for this parameter, reflecting a moderate dispersion.

Supplementary Information

Part III: Posterior distribution and model fit when using minimum and maximum estimates

The model presented in the main text of this article uses the mean values between the minimum and maximum estimates of test coverage and diagnosis rate from Chandra *et al.* [6] Here, we show the results for models 1-4 calibrated using the minimum and maximum estimates of tests and diagnoses separately. For example, yearly test coverage is defined as $\tau_{k,a,y} := \tau_{k,a,y}^{min}$ or $\tau_{k,a,y} := \tau_{k,a,y}^{max}$ for the minimum and maximum estimates, respectively, instead of using Equation 14. Tables S1 and S2 show the posterior parameter distributions in the models using the minimum and maximum estimates, respectively.

Table S1: Summary of parameters (mean and 95% CrI of posterior distributions) for different models. In these models we used the minimum estimates for tests and diagnoses from Chandra et al. [6] *for parameter inference. The last two rows show the fit statistics of the models. *Kept as fixed values in these models.*

	Model 1: posterior mean (95%CrI)	Model 2: posterior mean (95%CrI)	Model 3: posterior mean (95%CrI)	Model 4: posterior mean (95%CrI)
γ	0.85(0.82,0.89)	0.85(0.81,0.89)	0.84(0.81,0.88)	0.84(0.8,0.88)
χ^S	11.21(8.49,13.56)	11.18(8.71,13.61)	10.98(8.51,13.63)	11.12(8.65,13.69)
ω	0.73(0.7,0.77)	0.73(0.7,0.77)	0.74(0.7,0.77)	0.74(0.71,0.77)
β	0.58(0.54,0.63)	0.57(0.54,0.63)	0.83(0.74,0.93)	0.82(0.72,0.92)
ϵ	0.76(0.52,0.94)	0.79(0.54,0.96)	0.81(0.58,0.97)	0.85(0.66,0.98)
q_M	0.13(0.09,0.17)	0.1(0.05,0.15)	0.13(0.08,0.17)	0.1(0.05,0.15)
q_F	0.12(0.07,0.18)	0.09(0.02,0.17)	0.13(0.06,0.19)	0.1(0.02,0.17)
η_1	1.99(1.37,2.64)	4.19(1.85,7.8)	2.38(1.62,3.2)	4.7(2.34,7.91)
η_2	0*	0.97(0.52,1.54)	0*	0.88(0.45,1.27)
κ	0*	0*	0.75(0.58,0.9)	0.75(0.55,0.9)
Log likelihood	-694.54(-699.02,-691.56)	-693.53(-698.69,-690.43)	-682.48(-687.53,-679.23)	-681.12(-686.41,-677.73)
DIC	1400	1399	1372	1372

Table S2: Summary of parameters (mean and 95% CrI of posterior distributions) for different models. In these models we used the maximum estimates for tests and diagnoses from Chandra et al. [6] *for parameter inference. The last two rows show the fit statistics of the models. *Kept as fixed values in these models.*

	Model 1: posterior mean (95%CrI)	Model 2: posterior mean (95%CrI)	Model 3: posterior mean (95%CrI)	Model 4: posterior mean (95%CrI)
γ	0.86(0.82,0.89)	0.86(0.82,0.89)	0.84(0.8,0.88)	0.84(0.8,0.88)
χ^S	11.26(8.9,13.77)	10.96(8.44,13.4)	11.11(8.96,13.49)	11.01(8.65,13.38)

ω	0.73(0.7,0.77)	0.73(0.7,0.77)	0.74(0.71,0.78)	0.74(0.71,0.77)
β	0.62(0.58,0.67)	0.61(0.57,0.65)	0.84(0.76,0.93)	0.81(0.72,0.89)
ϵ	0.74(0.5,0.92)	0.82(0.61,0.97)	0.78(0.56,0.95)	0.82(0.59,0.97)
q_M	0.15(0.09,0.2)	0.08(0.02,0.15)	0.16(0.1,0.22)	0.12(0.05,0.19)
q_F	0.17(0.08,0.28)	0.11(0.01,0.23)	0.2(0.09,0.3)	0.16(0.05,0.26)
η_1	2.11(1.34,2.98)	6.78(3.71,9.61)	2.37(1.42,3.51)	5.66(2.14,9.71)
η_2	0*	1.27(0.87,1.51)	0*	0.84(0.15,1.42)
κ	0*	0*	0.69(0.51,0.86)	0.64(0.43,0.82)
Log likelihood	-715.26(-719.74,-712.42)	-709.46(-714.46,-706.27)	-701.81(-706.77,-698.83)	-698.58(-704.64,-694.75)
DIC	1443	1432	1411	1418

Part IV: Model fits for model 1-3

The model fits for the model with the lowest DIC (model 4) to prevalence data and data on diagnoses are shown in Figures 2 and 3, respectively, of the main text. Figures S1-S6 below show the model fits for models 1-3.

Supplementary Information

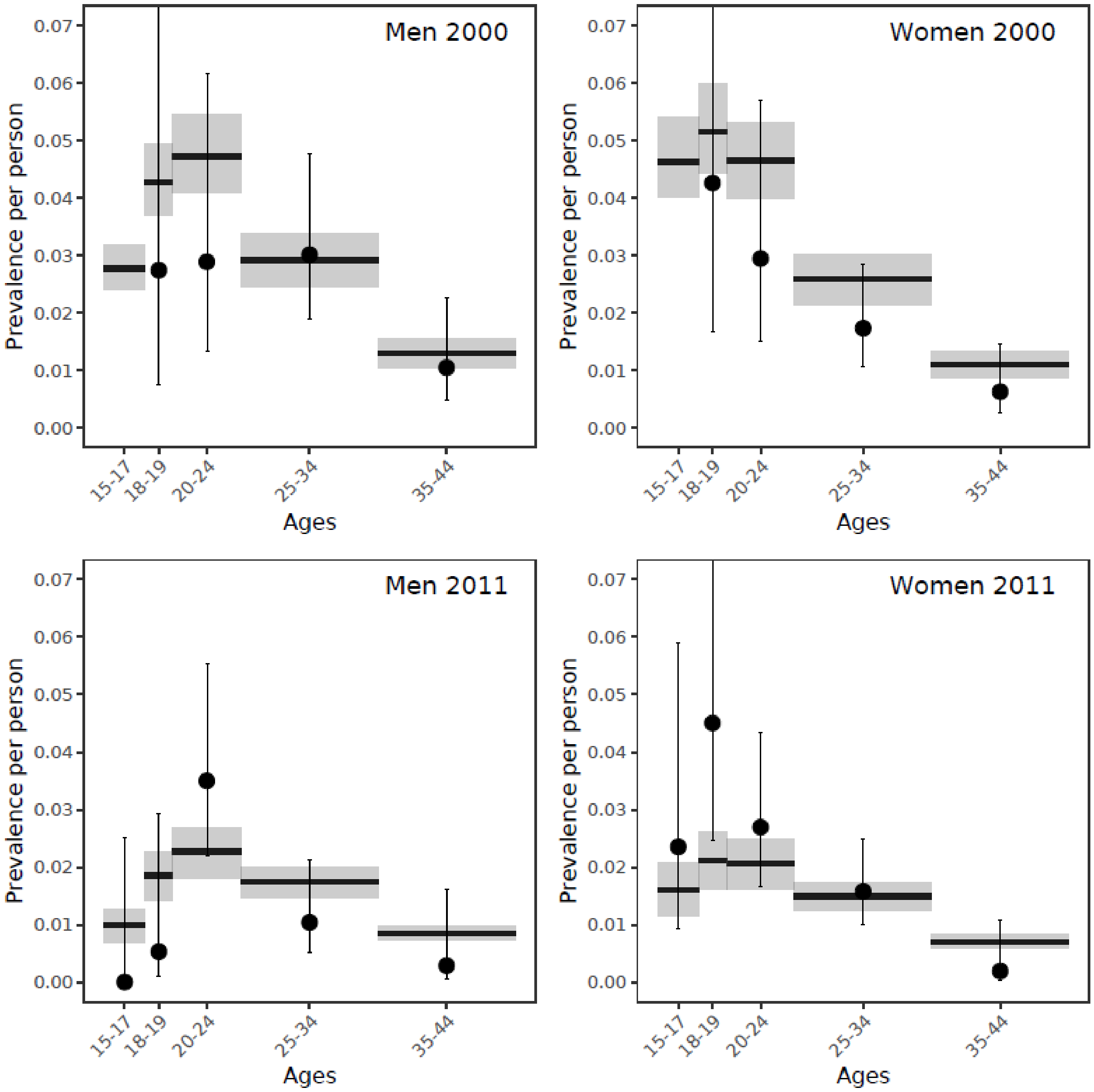

Figure S1: Fit of model 1 (excluding partial immunity and changes in the proportion of tests done in infected individuals) to age-specific chlamydia prevalence for men and women in 2000 and 2011. Grey boxes and horizontal lines: posterior mean and 95% Bayesian credible intervals. Black dots and vertical bars: Estimated prevalence from Natsal-2 (2000) and Natsal-3 (2011) (mean and 95% confidence intervals).

Supplementary Information

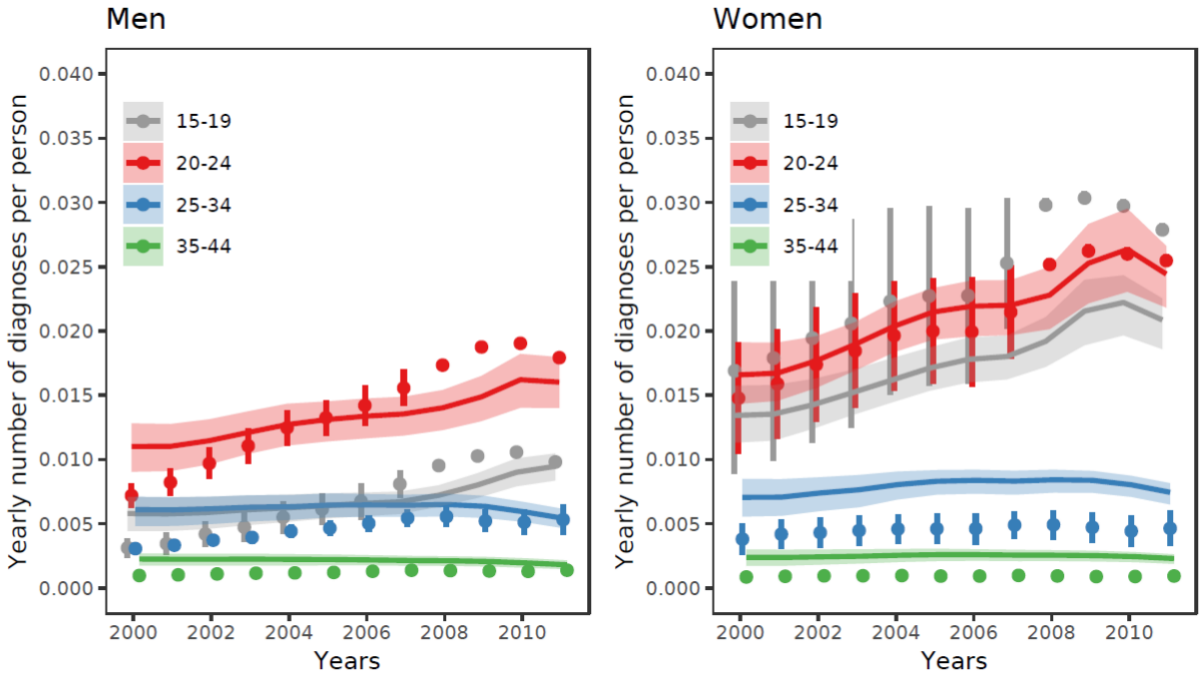

Figure S2: Fit of model 1 (excluding partial immunity and changes in the proportion of tests done in infected individuals) to age-specific per capita number of diagnoses for men and women between 2000 and 2011. Coloured lines and shaded areas: posterior mean and 95% Bayesian credible intervals. Vertical bars and dots: Minimum and maximum estimates for number of diagnoses from Chandra et al, and midpoints of these estimates (used for fitting).

Supplementary Information

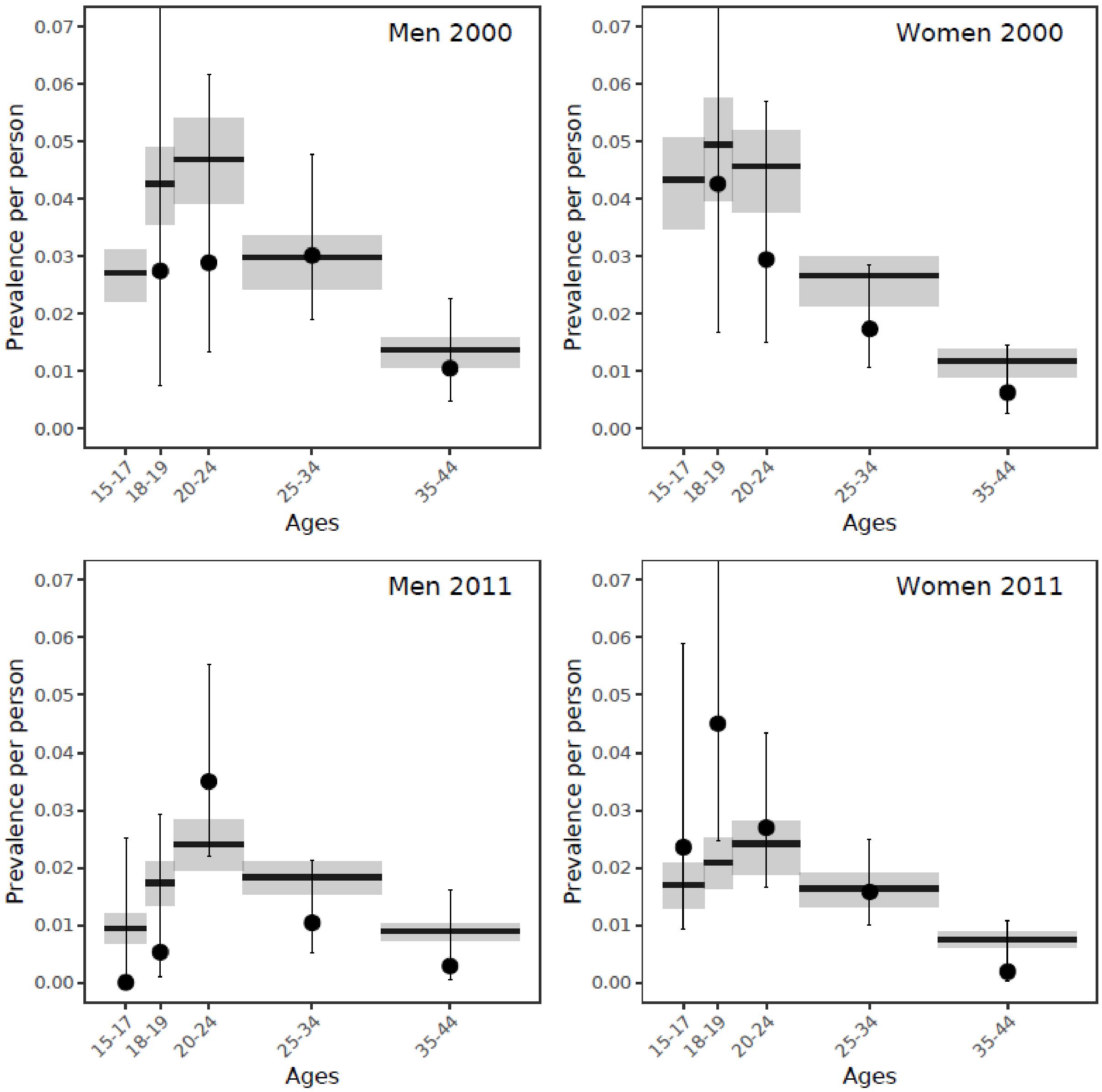

Figure S3: Fit of model 2 (including changes in the proportion of tests done in infected individuals; excluding partial immunity) to age-specific chlamydia prevalence for men and women in 2000 and 2011. Grey boxes and horizontal lines: posterior mean and 95% Bayesian credible intervals. Black dots and vertical bars: Estimated prevalence from Natsal-2 (2000) and Natsal-3 (2011) (mean and 95% confidence intervals).

Supplementary Information

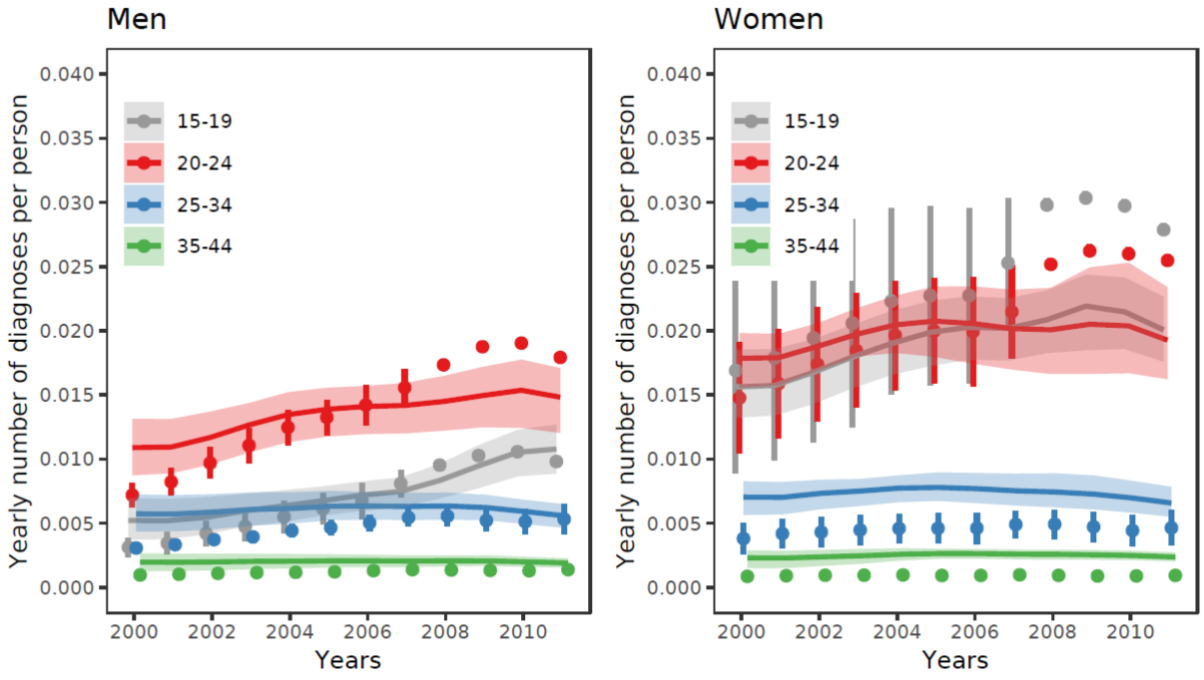

Figure S4: Fit of model 2 (including changes in the proportion of tests done in infected individuals; excluding partial immunity) to age-specific per capita number of diagnoses for men and women between 2000 and 2011. Coloured lines and shaded areas: posterior mean and 95% Bayesian credible intervals. Vertical bars and dots: Minimum and maximum estimates for number of diagnoses from Chandra et al, and midpoints of these estimates (used for fitting).

Supplementary Information

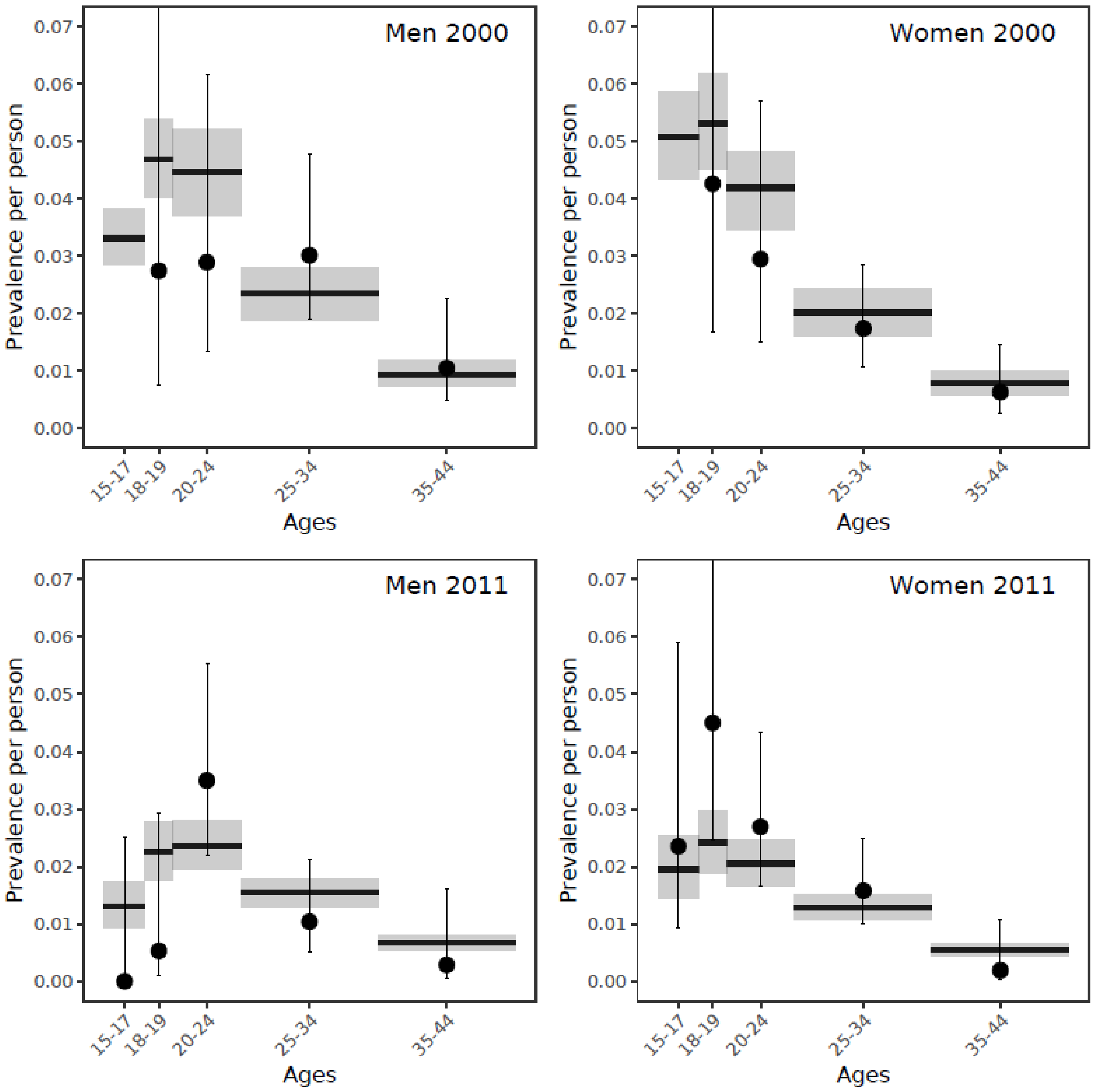

Figure S5: Fit of model 3 (including partial immunity; excluding changes in the proportion of tests done in infected individuals) to age-specific chlamydia prevalence for men and women in 2000 and 2011. Grey boxes and horizontal lines: posterior mean and 95% Bayesian credible intervals. Black dots and vertical bars: Estimated prevalence from Natsal-2 (2000) and Natsal-3 (2011) (mean and 95% confidence intervals).

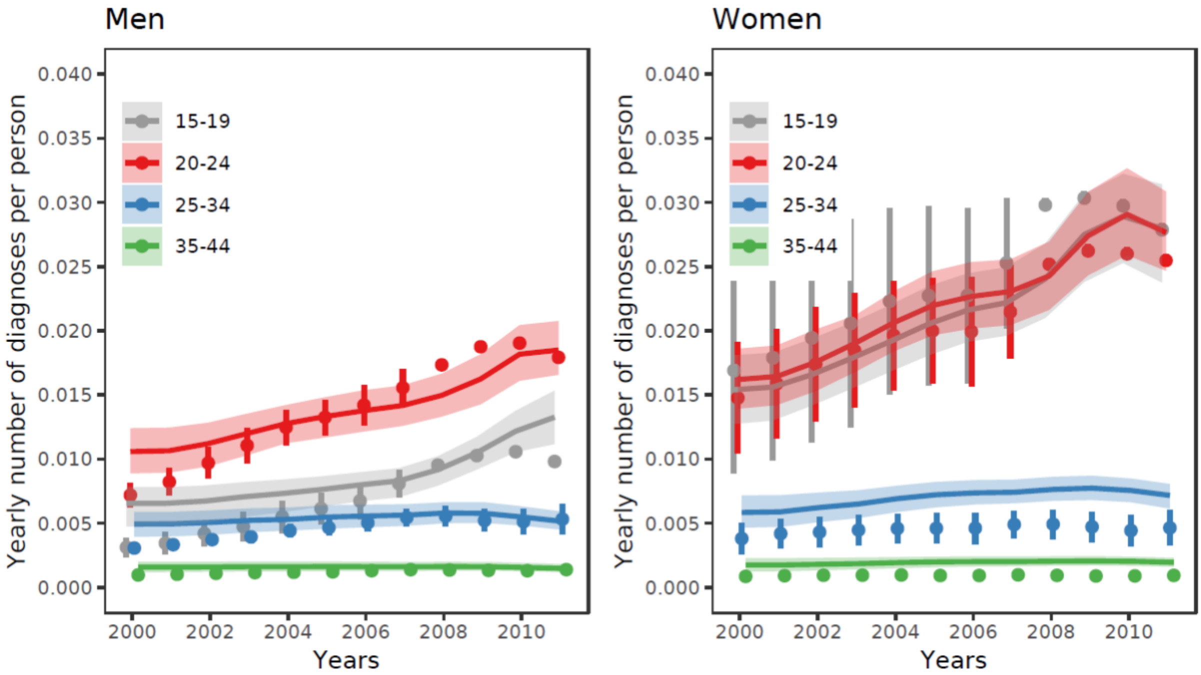

Figure S6: Fit of model 3 (including partial immunity; excluding changes in the proportion of tests done in infected individuals) to age-specific per capita number of diagnoses for men and women between 2000 and 2011. Coloured lines and shaded areas: posterior mean and 95% Bayesian credible intervals. Vertical bars and dots: Minimum and maximum estimates for number of diagnoses from Chandra et al, and midpoints of these estimates (used for fitting).

Part V: Additional figures

To improve understanding of what is happening in the model to testing patterns and indicators for chlamydia infection over time, we have provided additional figures for the full model (model 4). These show test coverage over time (Figure 7), the screening rates in asymptomatically infected people over time (Figure 8), the total screening rate over time (Figure 9), the differential screening coverage in 15-19 and 20-24 year old people over time (Figure 10), trends in prevalence (Figure 10), incidence (Figure 11) and positivity rate (Figure 12) over time.

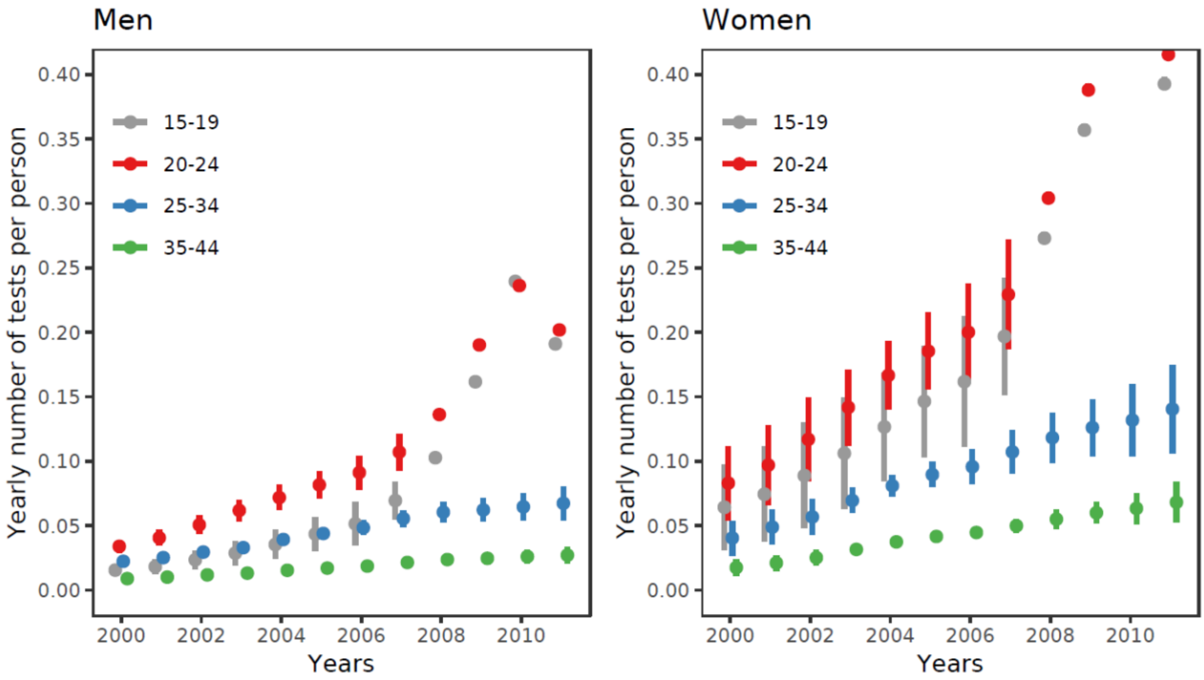

Figure S7: Testing rates per person per year from Chandra et al. The end points of the vertical bars represent the minimum and maximum estimates for number of tests between 2000 and 2011. The dots represent the midpoints of the minimum and maximum estimates, used in the model.

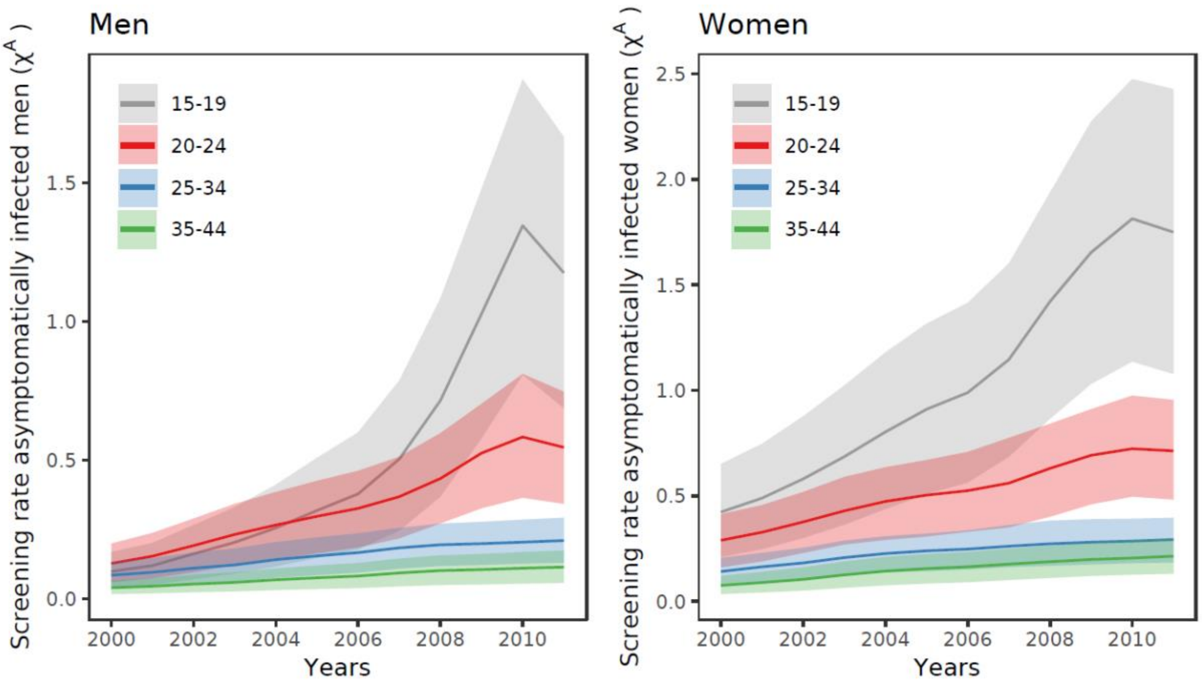

Figure S8: Model-estimated screening rates in asymptomatically infected men and women in different age groups between 2000 and 2011. Coloured lines and shaded areas: posterior mean and 95% Bayesian credible intervals

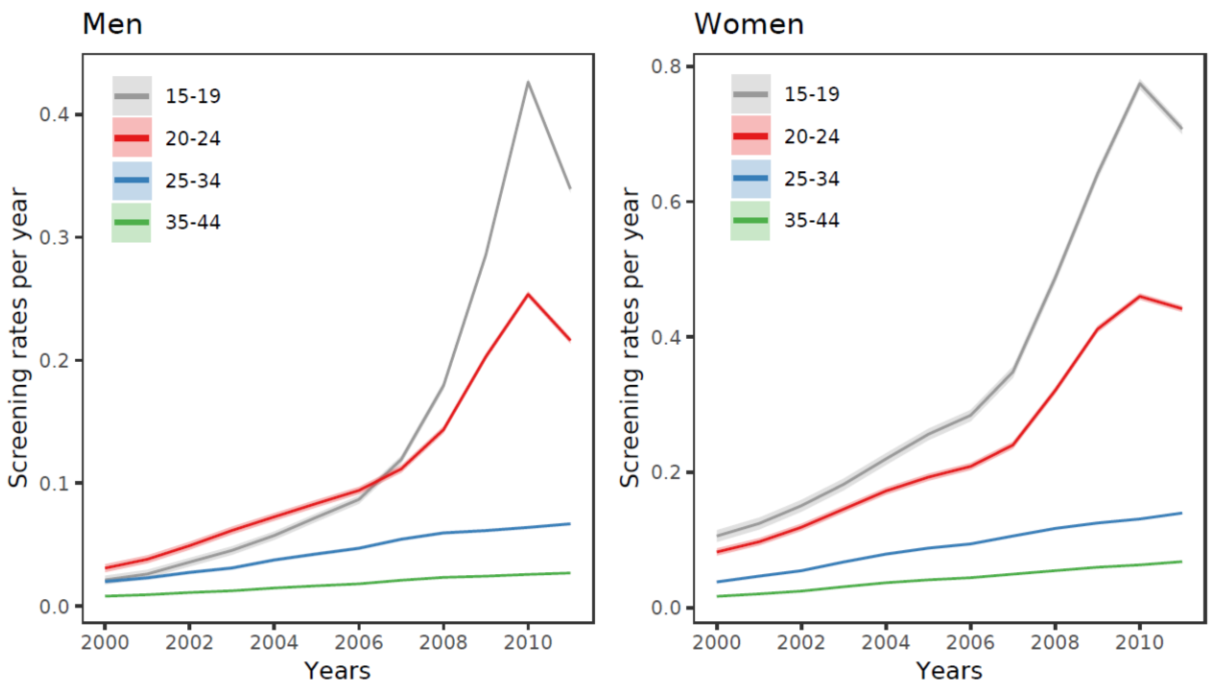

Figure S9: Model-estimated number of screening tests per person per year in all men and women in different age groups between 2000 and 2011. Coloured lines and shaded areas: posterior mean and 95% Bayesian credible intervals

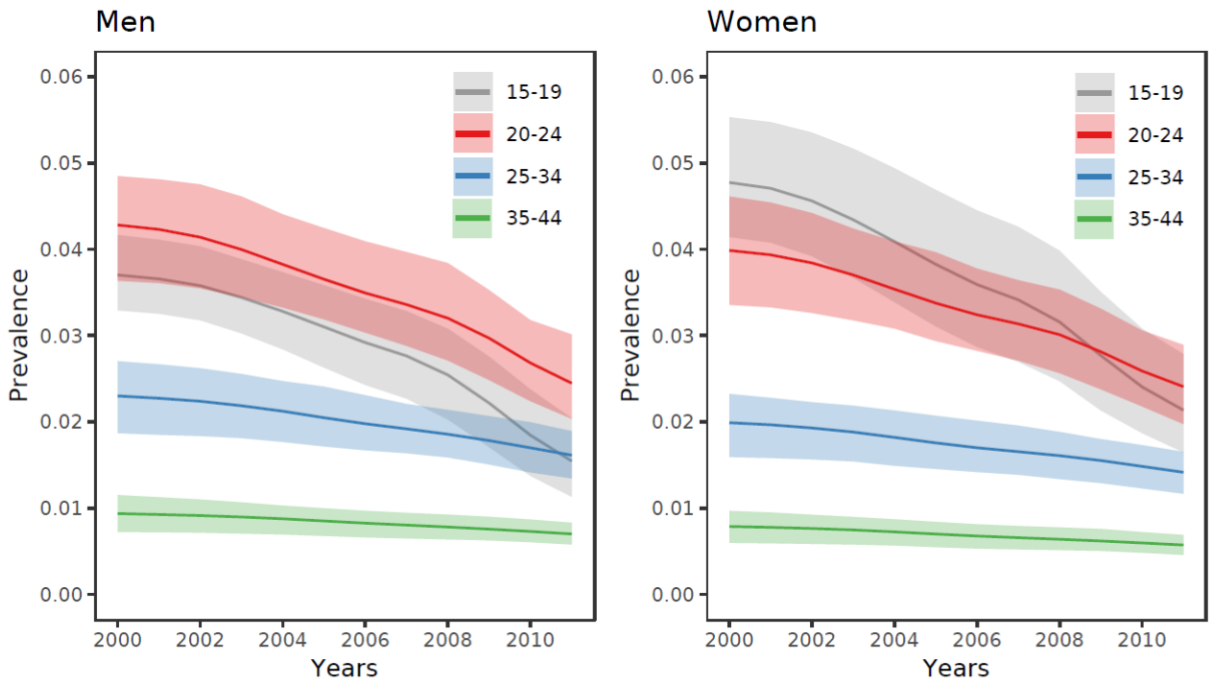

Figure S10: Model-estimated prevalence in men and women in different age groups between 2000 and 2011. Coloured lines and shaded areas: posterior mean and 95% Bayesian credible intervals

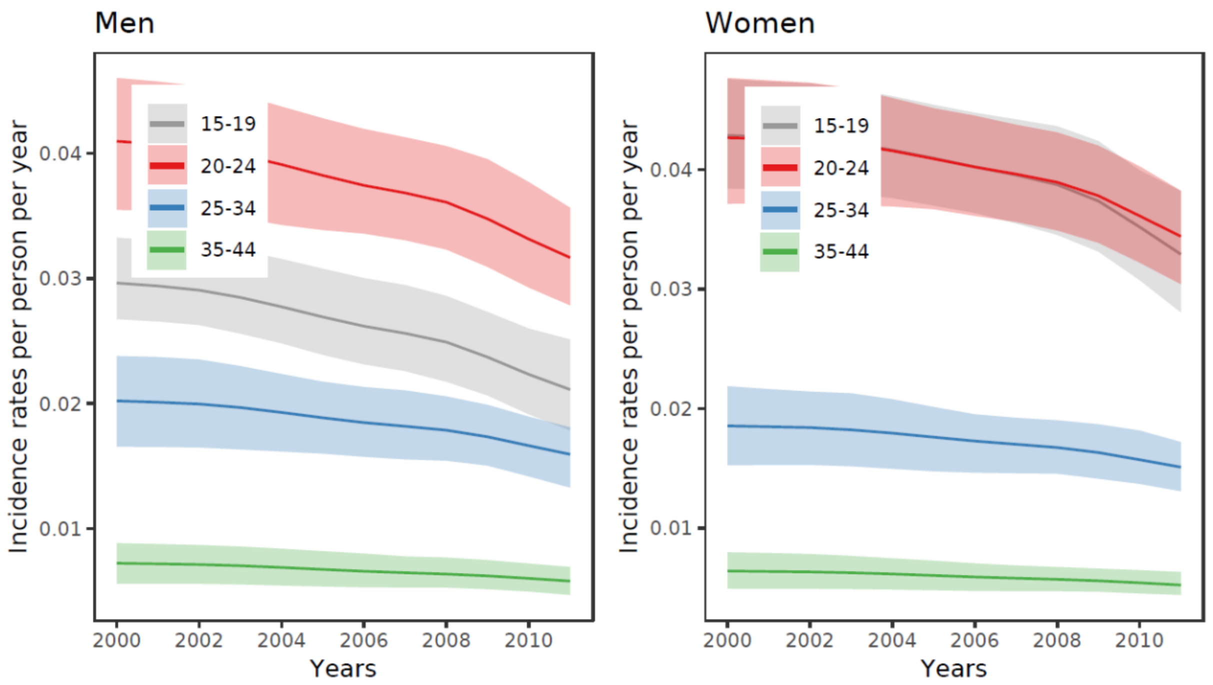

Figure S11: Model-estimated incidence rates per person per year in men and women in different age groups between 2000 and 2011. Coloured lines and shaded areas: posterior mean and 95% Bayesian credible intervals

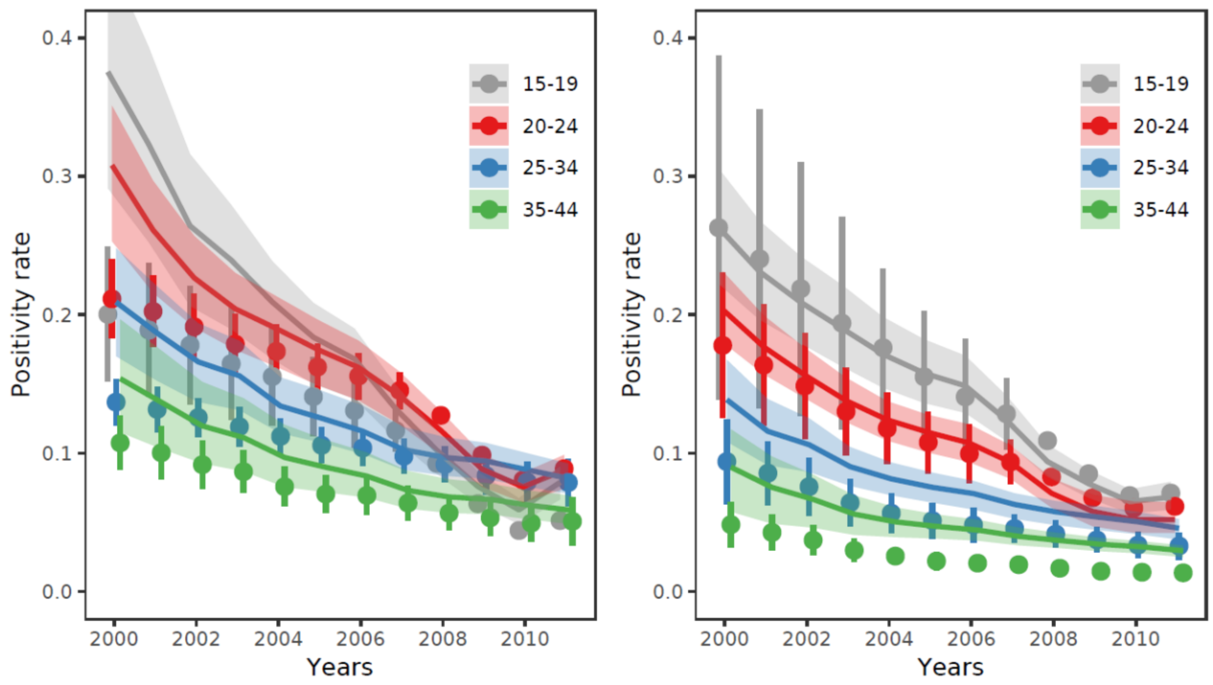

Figure S12: Positivity rates of chlamydia tests from Chandra et al, and model-estimates positivity rates for men and women in different age groups between 2000 and 2011. Coloured lines and shaded areas: posterior mean and 95% Bayesian credible intervals. Vertical bars and dots: Minimum and maximum estimates from data, and midpoints of these estimates. Positivity rates were computes by dividing the number of diagnoses in a sex and age stratum by the number of tests done in that stratum.

References

1 Fingerhuth, S. M., Bonhoeffer, S., Low, N. & Althaus, C. L. Antibiotic-Resistant Neisseria gonorrhoeae Spread Faster with More Treatment, Not More Sexual Partners. *PLoS Pathog* **12**, e1005611, doi:https://doi.org/10.1371/journal.ppat.1005611 (2016).

2 Garnett, G. P. & Anderson, R. M. Balancing sexual partnerships in an age and activity stratified model of HIV transmission in heterosexual populations. *IMA J Math Appl Med Biol* **11**, 161-192 (1994).

3 Fenton, K. A. *et al.* Sexual behaviour in Britain: reported sexually transmitted infections and prevalent genital Chlamydia trachomatis infection. *Lancet* **358**, 1851-1854, doi:https://doi.org/10.1016/S0140-6736(01)06886-6 (2001).

4 Sonnenberg, P. *et al.* Prevalence, risk factors, and uptake of interventions for sexually transmitted infections in Britain: findings from the National Surveys of Sexual Attitudes and Lifestyles (Natsal). *Lancet* **382**, 1795-1806, doi:https://doi.org/10.1016/S0140-6736(13)61947-9 (2013).

5 Smid, J. H., Garcia, V., Low, N., Mercer, C. H. & Althaus, C. L. Age difference between heterosexual partners: implications for the spread of Chlamydia trachomatis. *Epidemics-Neth* **24**, 60-66, doi:https://doi.org/10.1016/j.epidem.2018.03.004 (2018).

6 Chandra, N. L. *et al.* Filling in the gaps: estimating numbers of chlamydia tests and diagnoses by age group and sex before and during the implementation of the English National Screening Programme, 2000 to 2012. *Euro Surveill* **22**, doi:https://doi.org/10.2807/1560-7917.ES.2017.22.5.30453 (2017).

7 Chib, S. & Greenberg, E. Understanding the Metropolis-Hastings Algorithm. *Am Stat* **49**, 327-335, doi:https://doi.org/10.2307/2684568 (1995).

8 Bolker, B. *Ecological Models and Data in R, Ch. 4.5.1.3*. (Princeton University Press, 2008).
